# Urbach-Wiethe Syndrome and the Ophthalmologist: Review of the Literature and Introduction of the First Instance of Bilateral Uveitis

**DOI:** 10.1155/2012/281516

**Published:** 2012-07-31

**Authors:** Seyed-Mojtaba Abtahi, Farzan Kianersi, Mohammad-Ali Abtahi, Seyed-Hossein Abtahi, Arash Zahed, Hamid-Reza Fesharaki, Zahra-Alsadat Abtahi, Shahzad Baradaran, Mehdi Mazloumi, Saeed Naghiabadi

**Affiliations:** ^1^Faculty of Medicine, Isfahan University of Medical Sciences, Isfahan, Iran; ^2^Department of Otolaryngology, Faculty of Medicine, Isfahan University of Medical Sciences, Isfahan, Iran; ^3^Isfahan Eye Research Center (IERC), Feiz Hospital, Isfahan University of Medical sciences, Isfahan, Iran; ^4^Ophthalmology Ward, Feiz Hospital, Isfahan University of Medical Sciences, Isfahan, Iran; ^5^Medical Student's Research Center, Isfahan University of Medical Sciences, Isfahan, Iran; ^6^S.H.A. Research Center of Neurological-Ophthalmological Sciences (SHARNOS Co.), No. 9 Boroomand, Seyed-Alikhan, Chaharbagh Abbasi, Isfahan 81448-14581, Iran; ^7^Vice Chancellery for Research, Isfahan University of Medical Sciences, Isfahan, Iran; ^8^Jahad Daneshgahi, The University of Isfahan, Isfahan, Iran; ^9^Eye Research Center, Farabi Eye Hospital, Tehran University of Medical Sciences, Tehran, Iran

## Abstract

Patients suffering from Urbach-Wiethe syndrome (UWS), also known as lipoid proteinosis or hyalinosis cutis et mucosae, may have an ophthalmologist involved in the diagnosis and management of their disease. Along with moniliform blepharosis as a pathognomonic feature of the disease, an ophthalmologist may encounter other manifestations of UWS in any part of the eye such as cornea; conjunctiva; sclera; trabecular meshwork; iris/pupil; lens and zonular fibers; retina; nasolacrimal duct. This paper provides a review on the pathogenesis and the diverse ocular manifestations seen in UWS patients. Uncommon complications are discussed in this paper (glaucoma; dry eye and epiphora; complications of lens, retina, cornea; iris/pupil and conjunctiva). Moreover, a 27-year-old male UWS patient is described with bilateral diffuse anterior stromal iris atrophy, diffuse keratic precipitates; posterior subcapsular cataract; 1 + vitreous cell in anterior vitreous examination. This case was thought to be the first instance of bilateral uveitis associated with UWS. Overall, ophthalmologists may encounter diverse ocular complications accompanying this syndrome. They should be familiar with well-established ophthalmologic manifestations leading them to cooperate with other specialists in diagnosis and management of the disease.

## 1. Introduction

Urbach-Wiethe syndrome (UWS) (also known as lipoid proteinosis or hyalinosis cutis et mucosae) is an autosomal recessive disorder [1], first described by Urbach and Wiethe in 1929 [2]. This disorder is extremely rare. So far, no more than 300 patients aging from 6 months to 67 years have been diagnosed with UWS [3–5]. UWS has a strong tendency towards the white with no sex preponderance. It is more common among Europeans, especially the Dutch and Germans [6–8]. Mutations responsible for UWS are known to occur on ECM1 gene located on 1q21 [9]. Pathophysiology of this multisystem disorder mostly includes deposition of hyaline-like material in the body with a ubiquitous pattern that, however, may vary from individual to individual. The most involved parts are viscera, mucosa, vocal cords, and skin [3–7, 10]. 

UWS has a chronic but benign course with a generally favourable prognosis. The disease is not usually life threatening, though, severely diminishes the quality of life. To manage its complications, several medical specialties may be engaged.

Essentially, UWS is categorized as a dermatologic disease, presenting with diffuse skin infiltration and thickening. Skin lesions generally appear as nodules on face, lips, and also papules (at earlier stages) to hyperkeratotic and warty lesions (at later stages) on fingers joints, knees, elbows, perineum, scrotum, and axilla. Moreover, in hairy parts, lesions may lead to hair loss, for example, scalp baldness [3–5, 10–14]. 

Alternatively, otolaryngologists deal with UWS cases. This is mostly due to the most striking symptom of UWS namely; hoarseness resulting from vocal cord involvement that usually harbingers the onset of the disease in infancy and early childhood [1, 14]. 

Also, neurologists (for seizures, etc.), psychiatrists (for behavioural changes, e.g.), and ophthalmologists are involved in the diagnosis and management of the disease [4, 6, 11–14]. Ophthalmologists typically visit a UWS, patient for a pathognomonic feature of the disease, named moniliform blepharosis that may be, though not typically, the presenting symptom [6, 14]. In the literature, along with such a typical manifestation, other rare ophthalmic manifestations are reported with UWS [15]. Nonetheless, as far as we are aware, there are no reports describing bilateral uveitis. 

The purpose of this study was to (1) illustrate the clinical and paraclinical features of a patient who presented with the above-mentioned condition, (2) to provide a review on ophthalmic manifestations of UWS. and (3) to present an outline on the proposed pathophysiologic mechanisms of the condition.

## 2. Case History

A 27-year-old Persian male was admitted to our centre with the chief complaint of blurred vision. In history taking he was a peasant who suffered from hoarseness since early childhood but had never been medically examined for its cause. He had noticed gradual visual loss since the year before, without any history of red eye or ocular pain. He had no family history for any significant diseases in his parents or the two siblings. His parents were cousins. In physical examination, his height and weight were 167 cm and 71 kg, respectively. He had extensive baldness of the scalp. Multiple scars and thickening of the face and body skin were prominent. There was no past history of smoking or addiction. In neurology examination, mental status was normal and there was no positive history for any significant neurological condition. 

**Figure 1:**
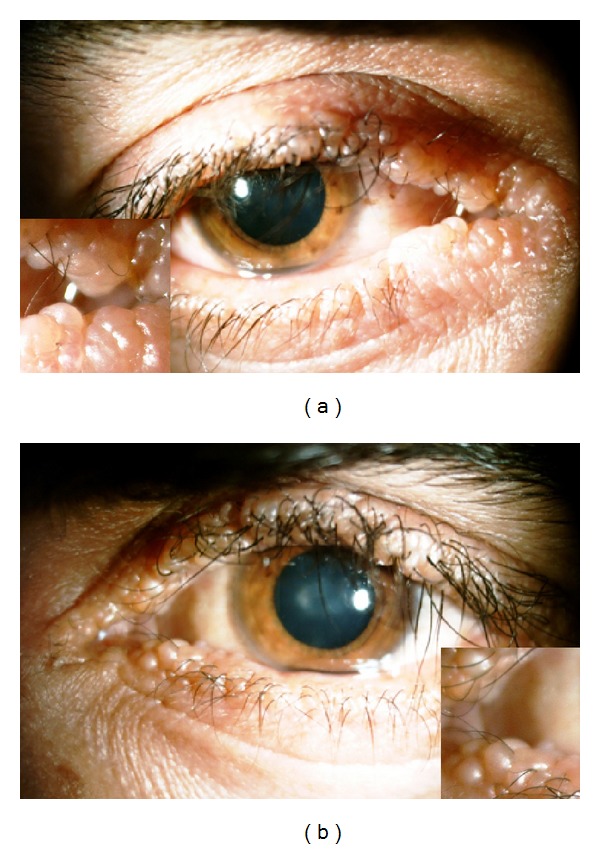
Moniliform blepharosis in (a) right and (b) left eye.

In ophthalmology examination, visual acuity was 20/100 OD and 20/200 OS. The presence of severe bilateral blepharitis and wart-like nodules on upper and lower eyelids was remarkable (moniliform blepharosis), though eyelashes were relatively intact (Figure 1).

**Figure 2:**
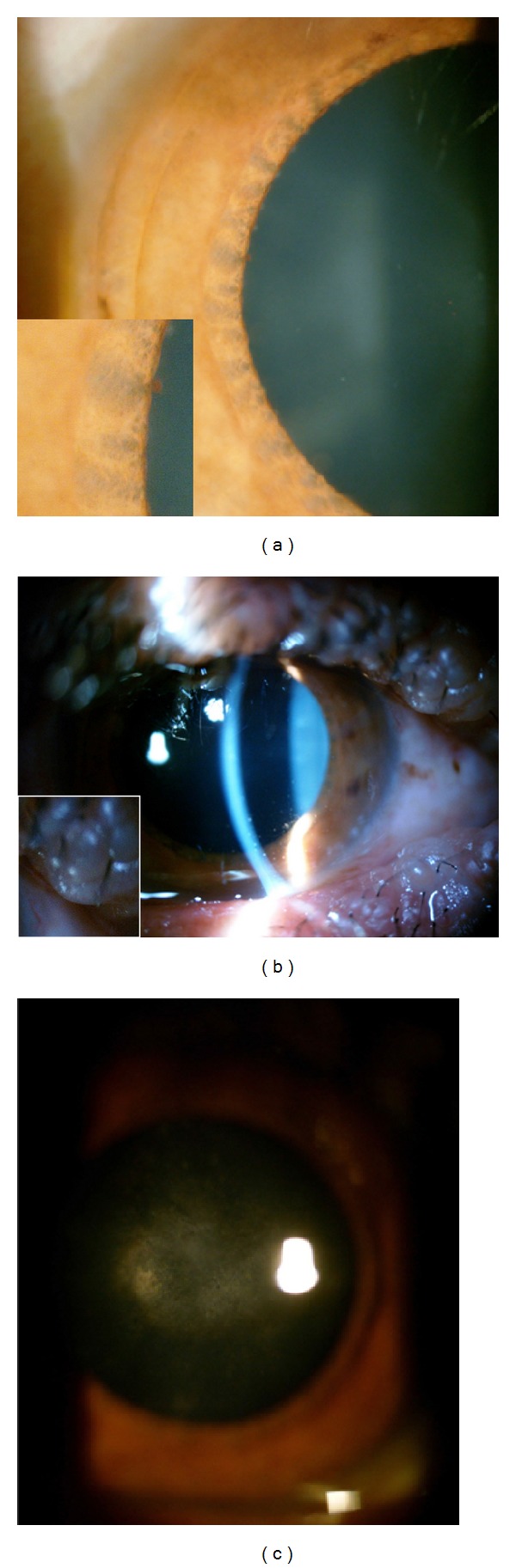
(a) Diffuse iris atrophy. (b) Diffuse keratic precipitates. (c) Posterior subcapsular cataract.

 Conjunctiva of both eyes was not hyperemic. Both eyes showed diffuse anterior stromal iris atrophy (Figure 2(a)), diffuse keratic precipitates (Figure 2(b)), and about 1 + flare. However, there was no evidence of posterior synechia formation. 

**Figure 3:**
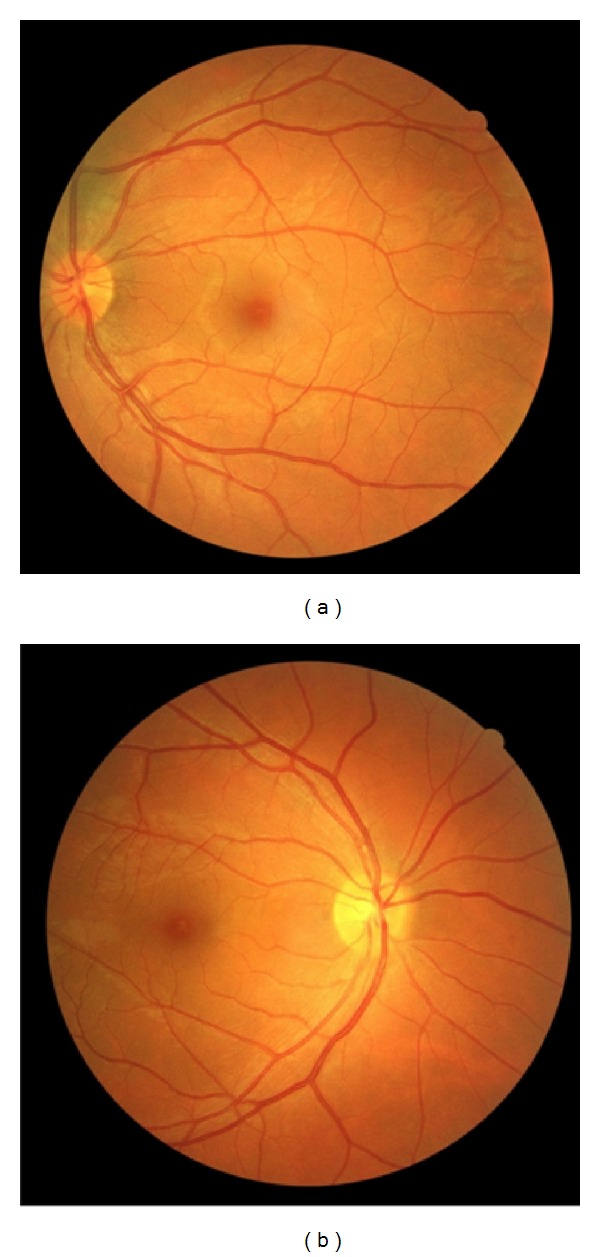
Fundus photographes of both eyes, one month after cataract surgery of the second eye. (a) Left eye. (b) Right eye.

Pupillary reactions to light and near stimuli were normal and no afferent pupillary defect was present. Intraocular pressure of both eyes was normal. Bilateral posterior subcapsular cataract was notable (Figure 2(c)), more severe in the left eye. Anterior vitreous examination showed 1 + vitreous cell in both eyes. Examination of retina (Figure 3) and ocular motility was unremarkable. Based on the above-mentioned observations, we confirmed the diagnosis of uveitis. 

General workup, including blood count, renal function test, urine analysis, blood sugar, blood chemistry, and chest X-ray, was performed and had no remarkable results. Also, hepatitis B surface antigen and hepatitis C antibody were negative. In rheumatologic consultation, tests for antinuclear antibody, rheumatoid factor, antineutrophil cytoplasmic antibody, angiotensin converting enzyme, purified protein derivative, and venereal disease research laboratory were carried out and all were negative.

**Figure 4:**
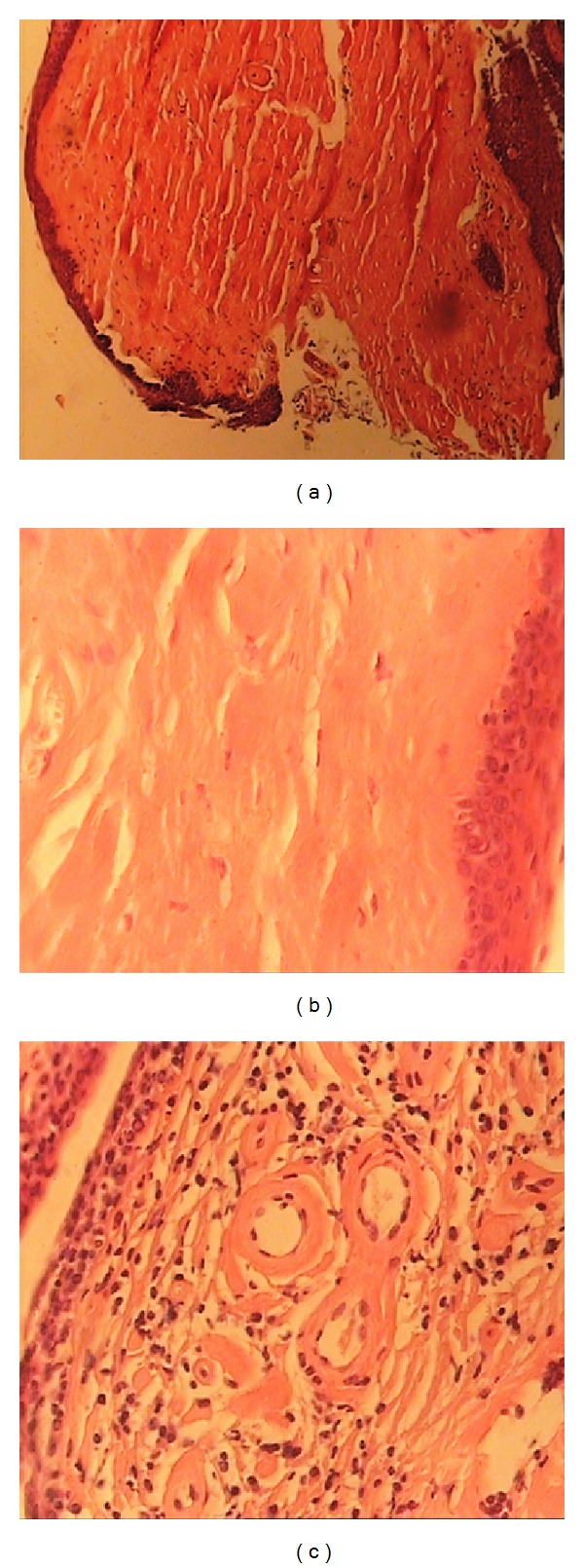
Biopsy of vocal fold (H&E): (a) and (b) deposition of hyaline material (×20 and ×400, resp.). (c) “Onion skin” appearance around blood vessels (×100).

To treat blepharitis, topical lubricant and antibiotic (tetracycline ointment) were administered. Because of no posterior synechia and active intraocular inflammation, we did not consider any further treatment for management of patient's uveitis. To evaluate the cause of hoarseness, we referred the patient to the Otolaryngology Department of our university hospital. Laryngoscopy showed thickening and irregularities on the patient's vocal cords. Biopsy of the vocal cords lesions revealed hyaline material deposition consistent with UWS (Figure 4). The diagnosis of UWS was finally confirmed by an otolaryngologist. 

The patient was scheduled for cataract surgery over the ensuing 4 weeks. He underwent an uncomplicated cataract surgery, first on his left eye and then on the contralateral eye with an interval of three weeks. An aqueous humor sample was collected during surgery and was studied using polymerase chain reaction for detection of varicella-zoster viruses, cytomegalovirus, and herpes simplex virus 1 and 2. The results of these tests were, however, negative. Three months after the operation, the visual acuity was better than 20/25 in both eyes and no progression of intraocular inflammation was detectable.

## 3. Discussion and Literature Review

In our case, the occurrence of UWS as an autosomal recessive disorder could be simply explained by the consanguinity of healthy parents. Interestingly, almost all previously reported cases of UWS had consanguine parents, and the most observed kinship in this regard was “first-cousin” which accords with our case [16]. 

UWS is not usually diagnosed by ophthalmologists. In general, its diagnosis is considered “often delayed and difficult” [13]. Consistently, in our case, the diagnosis was established when ocular symptoms became prominent several years after the onset of other dermatologic and otolaryngologic symptoms. In addition to the definite diagnosis of UWS, our findings leave no doubt that our case developed bilateral uveitis and posterior subcapsular cataract. Moreover, typical manifestation of moniliform blepharosis can be considered as another ophthalmologic complication in our patient.

Ocular signs/symptoms were described as a part of UWS, since early descriptions [17, 18]. As far as we could elicit from the literature, these manifestations may fall into two major groups: (1) common findings described in most patients and (2) rare findings reported in a single or few cases. Generally, involvement of different parts of the eye such as cornea, conjunctiva, sclera, trabecular meshwork, pupil/iris, lens/zonular fibers, retina, and nasolacrimal duct is reported [7, 12, 14, 15, 17, 18]. In spite of the diversity of these manifestations, limited theories have been put forward to explain their aetiology [15–17]. The vast majority of authors, who reported either common or uncommon ocular manifestation, explained the physiopathology of their observation by adhering to the simple fact that hyaline material would deposit in any part of the eye and in turn, gives rise to inflammation or abnormal functioning [14, 15, 17, 18]. Nonetheless, exceptional explanations have also been proposed [19, 20] which will be reviewed. 

### 3.1. Common Ocular Manifestations of UWS

Generally, eyelid lesions are reported in at least two-thirds of the cases [18]. As mentioned before, classic presentation of such lesions is called moniliform blepharosis, which is generally believed to be one of the most pathognomonic features of UWS [13, 14]. This hallmark presents as tiny papules on eyelid margins just like a string of yellowish and waxy beads (Figure 1) and is particularly known as a strong diagnostic clue [21–23]. Some authors, however, believed that these lesions “are hardly of any clinical significance” [6]. Apart from the diagnostic value, lid lesions are reported to accompany infiltration of glands of Zeiss, Moll, and Meibomian [7] and consequently cause madarosis, trichiasis, and sometimes distichiasis [15, 18]. Hewson [24] presented a male UWS case with ulceration of the right cornea caused by trichiasis. The ulcer healed with scarring and the visual acuity reduced to 6/36. Description of this case further highlighted the importance of examining the lid edges for ingrowing lashes in these patients. Later, some authors suggested that these lesions should be treated to prevent further complications. Main suggested modalities are surgical removal [14] and CO_2_ laser therapy (0.2 mm spots and W 1-2) [25]. 

Another frequent finding in UWS is focal degeneration of macula and drusen formation in Bruch's membrane which have been observed in a third to a half of the examined patients [18, 26]. In some patients with documented histo-pathological evaluations, thickening of Bruch's membrane, as well as partial infiltration of vessel walls of the choroid/retina by hyaline PAS positive material are described [6, 15, 18, 27–29]. 

### 3.2. Rare Ocular Manifestations of UWS

#### 3.2.1. Glaucoma

Zapata et al. described a UWS case with bilateral chronic open-angle glaucoma in 2004 and asserted to have presented the first instance of this condition [30]. However, in the older literature we could trace two other cases with open-angle glaucoma [18]. Besides, another instance of angle-closure glaucoma had been reported in 1961 by Marquardt [27]. The deposition of hyaline inclusions in the trabecular meshwork was suggested as the cause of open-angle glaucoma [12, 15, 18, 31]. Another relevant case with ocular hypertension was reported by Weybrecht and Korting in 1953. They postulated that this condition might be due to hyalinization of scleral trabeculum with deposition of glycoproteins [32].

#### 3.2.2. Lens-Related Complications

In a study in Egypt, a UWS patient with bilateral congenital cataract and myopic fundus was described. The case had some other characteristic features such as flat face, pinched nose, and fair hair. The authors proposed that their patient displayed a new autosomal recessive disease with concurrent skin and eye manifestations [33].

Our case presented with bilateral posterior subcapsular cataract. Accordingly, we could find another case with “bilateral nuclear lens opacifications” [34]. Another lens-related complication was reported by Mandal et al. [20], describing a 32-year-old man with bilateral crystalline lens subluxation. Their case was further complicated by anterior dislocation in the right eye and nasal subluxation in the contralateral eye. The authors hypothesized that defective lens zonular formation may be due to the underlying genetic mutations attributed to UWS. However, this hypothetical pathomechanism remains to be proven.

#### 3.2.3. Retinal Complications

In addition to common retinal manifestations noted above, the concomitancy of retinitis pigmentosa and UWS is noteworthy [35]. Moreover, in the literature, some authors addressed the association of UWS with impaired colour vision and light hypersensitivity [15].

#### 3.2.4. Corneal Complications

Specific corneal manifestations seem to occur more rarely. In addition to the aforementioned corneal ulceration caused by trichiasis [24], there are some notifications of corneal opacities as well as the deposition of hyaline on the cornea, especially at Descemet's membrane [12, 17].

#### 3.2.5. Iris- and Pupil-Related Complications

As in other parts, hyaline deposits can also be found on the iris [15]. More interestingly, we must mention a 32-year-old male who had had pupillary abnormalities since he was 9 months. In this patient, bilateral corectopia with displacement of the pupil superiorly and nasally was present. Authors postulated that the possible presence of hyalinised material in the rostral midbrain could have caused such a condition and also suggested the addition of UWS to the differential diagnosis of corectopia [19].

#### 3.2.6. Conjunctival Complications

Descriptions of granulations or infiltration of hyaline material in conjunctiva exist in the literature [15, 17]. We could also find some notes of a case with a small yellow nodule in the conjunctiva [36].

#### 3.2.7. Dry Eye and Epiphora

In a pair of consecutive studies, another category of symptoms was introduced as dry eye and epiphora in a brother [3] and sister [37], respectively. First, in 1996, Irkeç et al. [3] reported the occurrence of dry eye syndrome in early life of a boy whose meibomian glands were seemingly affected by the deposits. They postulated that UWS-related histopathologic changes might disturb the normal functioning of lacrimal gland and reduce tear production. Later in 2001 [37], another member of the family—a 6-year-old girl—was introduced with bilateral punctal stenosis and epiphora. The lacrimal puncta of both eyes were covered by cystic structures identified as the cause of epiphora. 

Based on our literature review, the above-mentioned siblings were not the only instances of dry eye or epiphora. Epiphora had been also reported in two sisters who were both definite cases of UWS [38]. On the other hand, “ocular dryness” was also reported to accompany xerostomia in a 59-year-old case of UWS [39].

#### 3.2.8. Nasolacrimal Duct Complications

Nasolacrimal duct obstruction is another entity observed in association with UWS. Two cases with this condition were described in the literature [40, 41]. More recently, Ostrovsky et al. described bilateral involvement of nasolacrimal ducts in an 80-year-old woman. Nevertheless, they did not detect any infiltration of hyaline deposits in the duct [40]. 

#### 3.2.9. Other

Juette in 1961, presented a case of UWS with calcification and thrombosis of the internal carotid artery which caused a transient blindness of the right eye [42].

#### 3.2.10. An Exceptional Case with Multiple Unilateral Complications

In the abovementioned sections, we reviewed particular cases with isolated ophthalmic manifestations ascribed to  UWS. In contrast to these cases, herein comes the review of a patient presented by Sellami et al. who displayed multiple one-sided ophthalmic complications. This case was a 28-year-old female presenting with hoarseness and cutaneous scars since early life. The diagnosis was confirmed by skin biopsy and laryngoscopy.

 At the age of 15, conjunctival hyperemia in the left eye became prominent and a yellowish perilimbal conjunctival infiltration appeared which was constructed of typical hyaline material in biopsy. Also she developed anterior chamber reaction with reduction of vision to 7/10. Afterwards, extension of conjunctival infiltration accompanying anterior chamber and vitreous cellular reaction occurred. Deposition of keratic precipitates and yellow iris nodules were also noted in this case. Later, the affected eye became functionally impaired to the level of light perception. This status was further complicated by posterior synechia, ocular hypertension, corneal edema, and cataract. Interestingly, until the end of followup, the contralateral (right) eye remained relatively spared [15]. 

#### 3.2.11. The First Instance of Bilateral Uveitis

To the best of our knowledge, concurrence of bilateral uveitis and UWS has never been described in the literature. The aforementioned exceptional patient of Sellami et al. is the only instance of unilateral uveitis in UWS who developed several overwhelming ophthalmic complications [15]. Comparatively, the presentation of uveitis in our case was bilateral and relatively isolated. The clinical picture of our case resembled to what is diagnosed as bilateral Fuchs heterochromic iridocyclitis (FHI). Typically, FHI is described as: little or no ciliary flush, characteristic white and stellate diffuse keratic precipitates, iris atrophy with or without heterochromia, posterior subcapsular lens opacities, and vitreous cells and debris [43–45]. Although, the features in our case were close to the clinical picture of FHI, we were unable to diagnose it with certainty. Since, FHI is typically a unilateral disorder (~90% of cases) with a different proposed aetiology [43–45]. The concurrence of bilateral uveitis and UWS in our case can be explained by what Sellami et al. proposed. They postulated that inflammatory response to iris damage or deposition of hyaline in the uvea might be responsible for the development of uveitis in UWS [15]. 

## 4. Conclusion

As can be understood from this paper, ophthalmologists may encounter diverse ocular complications associated with UWS. They should be familiar with ophthalmic symptoms/signs of the disease leading them to cooperate with other specialties in establishing a correct and timely diagnosis. Also, by improving their insight into other less common ocular complications, they can clearly play a more effective role in managing individuals with this disease.

## References

[1] Xu W, Wang L, Zhang L, Han D, Zhang L (2010). Otolaryngological manifestations and genetic characteristics of lipoid proteinosis. *Annals of Otology, Rhinology and Laryngology*.

[2] Urbach E, Wiethe C (1929). Lipoidosis cutis et mucosae. *Virchows Archiv für Pathologische Anatomie und Physiologie und für Klinische Medizin*.

[3] Irkec M, Orhan M, Orhan D, Durgun B, Can C (1996). Dry eye syndrome associated with Urbach-Wiethe disease. *Journal of Pediatric Ophthalmology and Strabismus*.

[4] Di Giandomenico S, Masi R, Cassandrini D (2006). Lipoid proteinosis: case report and review of the literature. *Acta Otorhinolaryngologica Italica*.

[5] Nasiri S, Sarrafi-Rad N, Kavand S, Saeedi M (2008). Lipoid proteinosis: report of three siblings. *Dermatology Online Journal*.

[6] Blodi FC, Whinery RD, Hendricks CA (1960). Lipoid-proteinosis (Urbach-Wiethe) involving the lids. *Transactions of the American Ophthalmological Society*.

[7] Fonseca EC, De Fendi LI, Andretta PS, Martin RT, Ottaiano JA (2007). Urbach-Wiethe syndrome: a case report. *Arquivos Brasileiros de Oftalmologia*.

[8] Rosenthal AR, Duke JR (1967). Lipoid proteinosis: case report of direct lineal transmission. *American Journal of Ophthalmology*.

[9] Hamada T, McLean WH, Ramsay M (2002). Lipoid proteinosis maps to 1q21 and is caused by mutations in the extracellular matrix protein 1 gene (ECM1). *Human Molecular Genetics*.

[10] Hofer PA, Ohman J (1974). Laryngeal lesions in Urbach Wiethe disease (lipoglycoproteinosis; lipoid proteinosis; hyalinosis cutis et mucosae). A histopathological and clinical study, including direct laryngoscopical examinations. *Acta Pathologica et Microbiologica Scandinavica A*.

[11] Santana N, Devi BK, Ramadoss T, Sumati T, Prasad S, Swamy S (2010). Lipid proteinosis: a case report. *Quintessence International*.

[12] Sainani MP, Muralidhar R, Parthiban K, Vijayalakshmi P (2011). Lipoid proteinosis of Urbach and Weithe: case report and a brief review of the literature. *International Ophthalmology*.

[13] Sharma V, Kashyap S, Betharia SM, Gupta S, Pathak H (2004). Lipoid proteinosis: a rare disorder with pathognomonic lid lesions. *Clinical and Experimental Ophthalmology*.

[14] Callizo M, Ibáñez-Flores N, Laue J, Cuadrado V, Graell X, Sancho JM (2011). Eyelid lesions in lipoid proteinosis or Urbach-Wiethe disease: case report and review of the literature. *Orbit*.

[15] Sellami D, Masmoudi A, Turki H (2006). Ophthalmic manifestations of lipoid proteinosis. *Presse Medicale*.

[16] Costagliola C, Verolino M, Landolfo P, Winkler NR, Mastropasqua L, Landolfo V (1999). Lipoid proteinosis (Urbach-Wiethe disease). *Ophthalmologica*.

[17] Deodati F (1964). The ophthalmological manifestations of Urbach-Wiethe lipoproteinosis. Apropos of a case. *Bulletin des Societes d'Ophtalmologie de France*.

[18] François J, Bacskulin J, Follmann P (1968). Ocular manifestations of the Urbach-Wiethe syndrome. Hyalitis of the skin and the mucosa. *Ophthalmologica*.

[19] Johnson LN, Hepler RS (1989). Corectopia and lipoid proteinosis. *The British Journal of Ophthalmology*.

[20] Mandal S, Dutta P, Venkatesh P, Sinha R, Kukreja M, Garg S (2007). Bilateral lens subluxation in a case of lipoid proteinosis. *Journal of Cataract and Refractive Surgery*.

[21] Thappa DM, Gupta S (2001). Eyelid beading—a useful diagnostic clue for lipoid proteinosis. *Indian Pediatrics*.

[22] Ofry V, Lewy A, Regenbogen L, Hanau D, Katznelson MB, Godel V (1979). Lipoid proteinosis (Urbach-Wiethe syndrome). *The British Journal of Ophthalmology*.

[23] Jensen AD, Khodadoust AA, Emery JM (1972). Lipoid proteinosis. Report of a case with electron microscopic findings. *Archives of Ophthalmology*.

[24] Hewson GE (1963). Lipidproteinosis (Urbach-wiethe syndrome). *The British Journal of Ophthalmology*.

[25] Rosenthal G, Lifshitz T, Monos T, Kachco L, Argov S (1997). Carbon dioxide laser treatment for lipoid proteinosis (Urbach-Wiethe syndrome) involving the eyelids. *The British Journal of Ophthalmology*.

[26] Bahadir S, Çobanoğlu Ü, Kapicioğlu Z (2006). Lipoid proteinosis: a case with ophthalmological and psychiatric findings. *Journal of Dermatology*.

[27] Marquardt R (1961). Eye changes in hyalinosis of the skin and mucous membrane (Urbach-Wiethe lipid proteinosis). *Ber Zusammenkunft Dtsch Ophthalmol Ges*.

[28] Gerth H, Flegel H (1956). Cutaneous and mucosal hyalinosis; symptomatology. *Dermatol Wochenschr*.

[29] Holtz KH, Schulze W (1951). Pathogenesis and clinical aspects of hyalinosis of the skin and mucosa (Urbach-Wiethe lipoid proteinosis). *Archives of Dermatology and Syphilology*.

[30] Zapata MA, Romera M, Linares F (2004). Juvenile glaucoma associated with syndrome of Urbach-Wiethe. Apropos of a case. *Annais d'Oftalmologia*.

[31] Huchzermeyer C, Ćirković A, Holbach L (2010). Micronodular thickening of eyelid margins.Initial findings of a general disease. *Ophthalmologe*.

[32] Weybrecht H, Korting GW (1954). Pathogenesis of cutis et mucosae Hyalinosis. *Archives of Dermatology and Syphilology*.

[33] Ashour AM, Temtamy SA, EI-Darouti M (2003). A probable new syndrome of lipoid proteinosis, congenital cataract and characteristic facies. *The Egyptian Journal of Medical Human Genetics*.

[34] Johnson LN, Hepler RS (1989). Corectopia and lipoid proteinosis. *The British Journal of Ophthalmology*.

[35] Schilovitz G, Grupper C, Payrau P (1973). Urbach-Wiethe disease. Association with retinitis pigmentosa. *Annales d'Oculistique*.

[36] Sanchez Caballero HJ, Ambrosetti FE, Lopez Lacarrere E (1954). Lipoido-proteinosis de Urbach. *La Semana Médica*.

[37] Orhan M, Mocan MC, Söylemezoğlu F, Irkeç M (2001). A case of epiphora associated with Urbach-Wiethe syndrome. *Eye*.

[38] Knorr HL, Meythaler HF, Naumann GO (1991). Epiphora as leading symptoms of Urbach-Wiethe syndrom in sisters. *Fortschritte der Ophthalmologie*.

[39] Disdier P, Harle JR, Andrac L, Swiader L, Weiller PJ (1994). Specific xerostomia during Urbach-Wiethe disease. *Dermatology*.

[40] Ostrovsky A, Mills DM, Farber M, Meyer DR (2007). Nasolacrimal duct obstruction with Urbach-Wiethe syndrome. *Ophthalmic Plastic and Reconstructive Surgery*.

[41] Gordon H, Gordon W, Botha V, Edelstein I (1971). Lipoid proteinosis. *Birth Defects Original Article Series*.

[42] Juette A (1961). Thrombosis of the internal carotid artery in hyalinosis of the skin and mucous membrane. *Ber Zusammenkunft Dtsch Ophthalmol Ges*.

[43] Velilla S, Dios E, Herreras JM, Calonge M (2001). Fuchs’ heterochromic iridocyclitis: a review of 26 cases. *Ocular Immunology and Inflammation*.

[44] Jones NP (1991). Fuchs’ heterochromic uveitis: a reappraisal of the clinical spectrum. *Eye*.

[45] Norrsell K, Sjödell L (2008). Fuchs’ heterochromic uveitis: a longitudinal clinical study. *Acta Ophthalmologica*.

